# Systematic Review of Evidence-Based Guidelines on Medication Therapy for Upper Respiratory Tract Infection in Children with AGREE Instrument

**DOI:** 10.1371/journal.pone.0087711

**Published:** 2014-02-20

**Authors:** Linan Zeng, Lingli Zhang, Zhiqiang Hu, Emily A. Ehle, Yuan Chen, Lili Liu, Min Chen

**Affiliations:** 1 Department of Pharmacy, West China Second University Hospital, Sichuan University, Sichuan, China; 2 Key Laboratory of Birth Defects and Related Diseases of Women and Children, Sichuan University, Ministry of Education, Sichuan, China; 3 Evidence-Based Pharmacy Centre, West China Second University Hospital, Sichuan University, Sichuan, China; 4 West China School of Pharmacy, Sichuan University, Sichuan, China; 5 Department of Pharmacy, The Nebraska Medical Centre, Omaha, Nebraska, United States of America; Kliniken der Stadt Köln gGmbH, Germany

## Abstract

**Objectives:**

To summarize recommendations of existing guidelines on the treatment of upper respiratory tract infections (URTIs) in children, and to assess the methodological quality of these guidelines.

**Methods:**

We searched seven databases and web sites of relevant academic agencies. Evidence-based guidelines on pediatric URTIs were included. AGREE II was used to assess the quality of these guidelines. Two researchers selected guidelines independently and extracted information on publication years, institutions, target populations, recommendations, quality of evidence, and strength of recommendations. We compared the similarities and differences of recommendations and their strength. We also analyzed the reasons for variation.

**Results:**

Thirteen guidelines meeting our inclusion criteria were included. Huge differences existed among these 13 guidelines concerning the categorization of evidence and recommendations. Nearly all of these guidelines lacked the sufficient involvement of stake holders. Further, the applicability of these guidelines still needs to be improved. In terms of recommendations, penicillin and amoxicillin were suggested for group A streptococcal pharyngitis. Amoxicillin and amoxicillin-clavulanate were recommended for acute bacterial rhinosinusitis (ABRS). An observation of 2–3 days prior to antibiotic therapy initiation for mild acute otitis media (AOM) was recommended with amoxicillin as the suggested first choice agent. Direct evidence to support strong recommendations on the therapy for influenza is still lacking. In addition, the antimicrobial durations for pharyngitis and ABRS were still controversial. No consensus was reached for the onset of antibiotics for ABRS in children.

**Conclusions:**

Future guidelines should use a consistent grading system for the quality of evidence and strength of recommendations. More effort needs to be paid to seek the preference of stake holders and to improve the applicability of guidelines. Further, there are still areas in pediatric URTIs that need more research.

## Introduction

Acute respiratory infections (ARIs) are classified as upper respiratory tract infections (URTIs) or lower respiratory tract infections (LRTIs) [1]. URTIs include the common cold, laryngitis, pharyngitis/tonsillitis, acute rhinitis, acute rhinosinusitis and acute otitis media (AOM) [2]. URTIs in children are a frequent illness accounting for a high proportion of doctor office visits [3], [4]. A national survey report from the UK showed the consultation rates of URTIs were 3,103 and 1,002 per 10,000 person years at risk in children aged 0–4 and 5–15 years, respectively [5].

A proliferation of clinical guidelines published in peer-reviewed journals has been seen due to the high morbidity of URTIs. It is important that these guidelines provide appropriate guidance for the treatment of URTIs. Nevertheless, the growing number of guidelines has been accompanied with a growing concern about variance and conflicts among guideline recommendations and the quality of guidelines [18].To date, there have been no systematic attempts to compare recommendations from available guidelines for the treatment of children with URTIs.

The aim of this study is to assess the quality of evidence-based guidelines for drug therapy of URTIs in children and to compare the recommendations of the existing evidence-based guidelines. Special attention was devoted to areas of disagreement and discussion with an ultimate aim to improve the clinical practice in treatment of URTIs for children. Such an assessment is important as it may explain some of the variability in guideline recommendations and may assist health care providers in choosing among available guidelines.

## Methods

### Data Sources

We searched Pubmed, Guidelines International Network (GIN), U.S. National Guideline Clearinghouse (NGC) and four Chinese databases: Chinese Biomedical Literature Database (CBM), China Knowledge Resource Integrated Database (CNKI), VIP Database and Wanfang Database for evidence-based guidelines (until March 2013) using the following items: respiratory tract infections, common cold, laryngitis, pharyngitis, tonsillitis, rhinitis, rhinosinusitis, otitis media, middle ear inflammation, influenza, grippe as Medical Subject Headings (MeSH) or keywords. The searches were limited to guidelines published in English or Chinese. We also searched guidelines at web sites of academic agencies, such as American Academy of Pediatrics (AAP) and Infectious Diseases Society of America (IDSA). Retrieved references were considered if they met our inclusion criteria.

### Guideline Selection

#### Inclusion criteria

Types: evidence-based guideline with systematic literature review and grading system for quality of evidence and/or strength of recommendation [6].

Diseases: URTIs including rhinosinusitis, pharyngitis, laryngitis, rhinitis, otitis media, tonsillitis, common cold and influenza.

Patients: children ages 0–18 years old

Interventions: drug therapy

#### Exclusion criteria

Types: guideline of hospital level; old version of guideline

Diseases: non-infectious upper respiratory diseases

Interventions: vaccines

### Guideline Quality Assessment

#### Appraisal of guidelines with the AGREE instrument

Quality of evidence-based guidelines was assessed by using AGREE II from the following domains: scope and purpose, stakeholder involvement, rigor of development, clarity of presentation, applicability, editorial independence and overall guideline assessment. Each of the AGREE II items and the two global rating items were rated on a 7-point scale (1-strongly disagree to 7-strongly agree). A score was assigned depending on the completeness and quality of reporting. Domain scores were calculated by summing up all the scores of the individual items in a domain and by scaling the total as a percentage of the maximum possible score for that domain. The scaled domain score was calculated as: (obtained score-minimum possible score)/(maximum possible score-minimum possible score) [7].

#### Appraisal of agreement between reviewers

We used the intraclass correlation coefficient (ICC) as a measure of agreement between reviewers. The ICC was applied to each guideline. Calculations were carried out by using SPSS 13.0.

### Data Extraction

Two researchers selected guidelines independently and extracted the following information: publication years, institutions, target populations, recommendations, quality of evidence, and strength of recommendations. We compared the similarities and differences of recommendations and their strength and analyzed the reasons for variation.

## Results

### Guideline Search and Review Process

**Figure 1:**
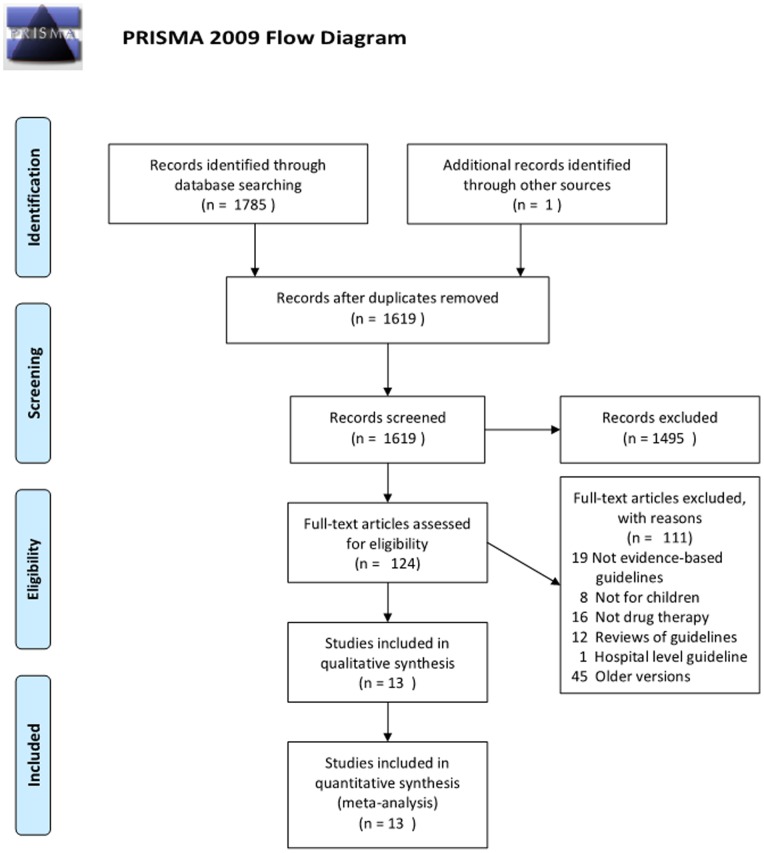
Summary of guideline search and review process.

A total of 1,785 citations and abstracts were identified in the initial searches. Finally, 13 guidelines meeting our inclusion criteria were included, covering a period from 2005 to 2013 (Figure 1). These 13 guidelines focused on drug therapy for pharyngitis, rhinosinusitis, otitis media and influenza. For the remaining URTIs (laryngitis, tonsillitis, rhinitis), no guidelines for children were found.

### Characteristics and Quality of Guidelines

Half of the 13 guidelines are from America developed by the American Academy of Pediatrics (AAP) and the Infectious Diseases Society of America (IDSA). Five guidelines are developed specially for children, while the rest are for both adults and children. All guidelines, except one, announced the conflict of interests and none were founded by industrial partners. All guidelines stated the formulation of recommendations was based on evidence. However, there is a huge variation in the grading systems of evidence quality and recommendation strength used (Table 1).

**Table 1:** Characteristics of 13 Evidence-Based Guidelines.

Guidelines by Medical Condition	Country	Institution^1^	Target Population	Conflicts of Interest^2^	Method to Formulate Recommendations	Quality of Evidence^3^	Strength of Recommendations^4^		Reference
**Pharyngitis**									
Shulman 2012	America	IDSA	Adult and child	SCI	Consensus development based on evidence	GRADE	GRADE		[8]
**Rhinosinusitis**									
Blomgren 2005	Finland	FSP	Adult and child	FPO	Consensus development based on evidence	Grading system from Evidence-based Medicine Working Group (A–D)		NA	[9]
Esposito 2008	Italy	SIP	child	EI	Consensus development based on evidence	Self designed grading system in accordance with the Italian National Guidelines Plan(I–VI)		Self designed grading system in accordance with the Italian National Guidelines Plan (A–E)	[10]
Chow 2012	America	IDSA	Adult and child	SCI	GRADE	GRADE		GRADE	[11]
Wald 2013	America	AAP	child	SCI	BRIDGE-Wiz^5^	Self designed grading system in accordance with the AAP policy statement (A–D,X)		Self designed grading system in accordance with the AAP policy statement “Classifying Recommendations for Clinical Practice Guidelines” (Strong recommendation, recommendation, option)	[12]
**Influenza**									
Bellamy 2006	–	WHO	Adult and child	SCI	GRADE	GRADE	GRADE		[13]
Bautista 2009	–	WHO	Adult and child	SCI	Consensus development based on evidence	GRADE	GRADE		[14]
Harper 2009	America	IDSA	Adult and child	SCI	Consensus development based on evidence	CTFPHE	CTFPHE		[15]
Morciano 2009	Italy	SNLG	Adult and child	FPO	Based on systematic review of available evidence	Self designed grading system in accordance with the Italian National Guidelines Plan(I–VI)	Self designed grading system in accordance with the Italian National Guidelines Plan (A–E)		[16]
**Otitis media**									
Bain 2003	England	SIGN	child	EI	Based on systematic review of available evidence	SIGN	SIGN		[17]
Takahashi 2012	Japan	JOS	child	NA	Based on available data	Grading system from Japan Stroke Society (I–IV)	Grading system from the US Preventive Services Task Force report (A–E)		[18]
Lieberthal 2013	America	AAP	child	SCI	Consensus development based on evidence	Self designed grading system in accordance with the AAP policy statement (A–D,X)	Self designed grading system in accordance with the AAP policy statement “Classifying Recommendations for Clinical Practice Guidelines” (Strong recommendation, recommendation, option)		[19]
**URTIs** 6									
Snellman 2013	America	ISCI	Adult and child	EI	Based on evidence summaries	Self designed grading system In transition to GRADE (High, Moderate, Low)	NA		[20]

Notes:

1 IDSA, Infectious Diseases Society of America; FSP, The Finnish Society of Otorhinolaryngology; SIP, Italian Society of pediatrics; AAP, American Academy of Pediatrics; WHO, World Health Organization; SNLG, Italian National Guidelines System; SIGN, Scottish Intercollegiate Guidelines Network; JOS, Japan Otological Society; ICSI, Institute for Clinical Systems Improvement; UMHS,University of Michigan Health System.

2 EI, editorial independence declared; FPO, funding by external public organization reported; SCI, statement about conflicts of interest of group members present.

3 GRADE, Grading of Recommendations Assessment, Development and Evaluation; CTFPHE, Canadian Task Force on the Periodic Health Examination.

4 NA: Not available.

5 An interactive software tool that leads guideline development through a series o f questions that are intended to create a more actionable set of key action statements.

6 URTIs refer to guidelines which include multiple diseases in URTIs.

### Comparison of the Categorization of Evidence and Recommendations of the 13 Guidelines (Table 2)

There were huge differences among 13 guidelines concerning the categorization of evidence and recommendations. Eight different grading systems were used, two of which failed to give strength of recommendations. Four guidelines used GRADE [21]–[25]. One guideline used SIGN [26] and one used CTFPHE [27]. Seven guidelines used other grading systems. The variation in terms of grading system may decrease the comparability of guidelines and confuse readers.

**Table 2:** Comparison of the categorization of evidence and recommendations of 13 guidelines.

Levels	CTFPHE	SIGN	GRADE	Self designed grading system In transition to GRADE^2^	Grading system from Evidence-based Medicine Working Group	Grading system from AAP	Grading system from Japan Stroke Society	Grading system from Italian National Guidelines System
**Quality of evidence**								
**1**	**I**: Evidence from ≥1 properly RCT^1^	**1^++^**:High quality meta-analyses, SR of RCTs, RCTs with a very low risk of bias.	**High quality**: Further research is very unlikely to change our confidence in the estimate of effect.	**High Quality Evidence:** Further research is very unlikely to change our confidence in the estimate of effect.	**A:**Several relevant, high-quality scientific studies with homogeneous results	**A:**Well-designed RCTs or DS on relevant population	**Ia**: Meta-analysis (with homogeneity) of RCTs	**I**: multiple RCTs and/or SR of randomized studies
		**1^+^**: Well conducted meta-analyses, SR, RCTs with a low risk of bias.					**Ib**: At least one RCT	
		**1** ^−^: Meta-analyses, SR, RCTs with a high risk of bias						
**2**	**II**: Evidence from ≥1 well-designed clinical trial, without randomization; from cohort or case-controlled analytic studies; from multiple time-series; from dramatic results from uncontrolled experiments	**2^++^**:High quality SR of case control or cohort studies; High quality case control or cohort studies with a very low risk of confounding or bias and a high probability that the relationship is causal.	**Moderate quality**: Further research is likely to have an important impact on our confidence in the estimate of effect and may change the estimate.	**Moderate Quality Evidence**: Further research is likely to have an important impact on our confidence in the estimate of effect and may change the estimate.	**B**:At least 1 relevant, high-quality study or several adequate studies	**B**:RCT or DS with minor limitations; overwhelmingly consistent evidence from OS	**IIa**: At least one well-designed, controlled study but without randomization.	**II**: one single adequate randomized trial
		**2^+^**: Well conducted case control or cohort studies with a low risk of confounding or bias and a moderate probability that the relationship is causal.					**IIb**: At least one well-designed, quasi-experimental study.	
		**2^−^**:Case control or cohort studies with a high risk of confounding or bias and a significant risk that the relationship is not causal						
**3**	**III:** Evidence from opinions of respected authorities, based on clinical experience, descriptive studies, or reports of expert committees	**3:**Non-analytic studies, eg case reports, case series.	**Low quality**: Further research is very likely to have an important impact on our confidence in the estimate of effect and is likely to change the estimate.	**Low Quality Evidence:** Further research is very likely to have an important impact on our confidence in the estimate of effect and is likely to change the estimate or any estimate of effect is very uncertain.	**C**:At least 1 adequate scientific study	**C**:OS	**III**: At least one well-designed, non-experimental descriptive study	**III**: non-randomized cohort, concurrent or historical studies, or their meta-analysis
**4**		**4:** Expert opinion	**Very low quality:** Any estimate of effect is very uncertain.		**D**:Expert panel evaluation or other information	**D:**Expert opinion, case reports, or reasoning from first principles	**IV**:Expert committee reports, opinions and/or experience of Respected authorities	**IV**:retrospective case-control studies
**5**						X: Exceptional situations in which validating studies cannot be performed and there is a clear preponderance of benefit or harm.		**V**: non-controlled case-series studies
**6**								**VI**: Expert opinion or opinions from panels as indicated in guidelines or consensus conferences
Strength of recommendation								
**1**	**A:** Good evidence to support a recommendation for or against use	**A:** rated as 1^++^ or 1^+^,and directly applicable to the target population	**strong recommendation:** the desirable effects of an intervention clearly outweigh the undesirable effects, or clearly do not	NA	NA	**Strong Recommendation:** quality of evidence is excellent benefits strongly outweigh the harms	**Strongly recommended:** strong evidence is available, benefits substantially outweigh harms.	**A:** the specified strongly recommended and based on good quality scientific evidence
**2**	**B:** Moderate evidence to support a recommendation for or against use	**B:** rated as 2^++^,and directly applicable to the target population; or Extrapolated evidence from studies rated as 1^++^ or 1^+^	**weak recommendation: **the trade-offs are less certain–either because of low quality evidence or because evidence suggests that desirable and undesirable effects are closely balanced			Recommendation:quality of evidence is not as strong; benefits exceed the harms	Recommended: fair evidence is available, benefits outweigh harms.	**B:** There are doubts as to whether the particular procedure should be always be recommended, but should be carefully considered.
**3**	**C:** Poor evidence to support a recommendation	**C:** Evidence level 3 or 4; or Extrapolated evidence from studies rated as 2^+^				**Option:** suspect evidence or well-done studies but little clear advantage to one approach vs another	**No recommendation made:** fair evidence is available, but the balance of benefits and harms is close.	**C:** There is substantial uncertainty concerning the procedure or intervention.
**4**						**No recommendation:** lack of evidence and an unclear balance between benefits and harms.	**Recommended against:** harms outweigh benefits.	**D:** the specified procedure is not recommended
**5**							**Insufficient evidence** to determine the balance of benefits and harms.	**E:** the specified procedure is strongly advised against.

Notes:

1 RCT: randomized controlled trials; DS: diagnostic studies; OS: Observational studies.

2 All existing ICSI Evidence Grading System incorporating GRADE methodology and all new literature considered by the work group for this revision has been assessed using GRADE methodology.

### Evaluation of the AGREE Domains of Guidelines Analyzed (Table 3)

#### Scope and purpose

This domain evaluates the overall objectives, expected benefit or outcomes, and target population of guidelines. The medium score for this domain was 90.28% (76.39%–97.22%), indicating that most guidelines satisfied criteria of this domain.

#### Stake holder involvement

This domain evaluates the degree of relevant professional group involvement and whether the views and preferences of the target population have been considered and the definition of target users has been clearly presented. The overall score in this domain was low with a medium of 61.11% (33.33%–83.33%). Most of the guidelines involved relevant professionals in the development process and declared the target population. However, the guideline developers did not seek the preference of target populations sufficiently resulting in a decrease in score of this domain.

#### Rigour of development

This domain addresses the method of evidence search, grading, summarizing and the formulation of recommendations. The medium score for this domain was 76.04% (32.81%–91.15%), with 1 guideline scoring <50%. This guideline failed to demonstrate the link between evidence and recommendations. It was not reviewed externally before its publication either.

#### Clarity of presentation

This domain evaluates presentation and format of guidelines. The medium score was 95.83% (90.28%–98.61%), indicating that all guidelines satisfied criteria of this domain.

#### Applicability

This domain evaluates the consideration of facilitators or barriers to its implementation, as well as monitoring criteria. The medium score of this domain was 56.25% (10.42%–83.22%), the lowest of all domains. Four of 13 guidelines scored ≤50%. Most guidelines failed to consider the applicability sufficiently in guideline development.

#### Editorial independence

This domain addresses founding issues and competing interests of guideline development members. The medium score was 81.25% (8.33%–97.92%), with 3 guidelines scoring<50%.

#### Agreement among reviewers

Table 3 summarizes the degree of agreement for 13 guidelines by ICC. The ICC values indicate overall agreement between reviewers was excellent (80%) for 8 of 13 guidelines and substantial (70%) for the other 5 guidelines.

**Table 3:** Quality Assessment by AGREE II of 13 Evidence-based Guidelines.

Guidelines by Medical Condition, y	Scores,%						Agreement among reviewers for AGREE instrument items	Overall Assessment^1^
	Domain 1 Scope and Purpose	Domain 2 Stakeholder Involvement	Domain 3 Rigour of Development	Domain 4 Clarityof Presentation	Domain 5 Applicability	Domain 6 Editorial Independence		
**Pharyngitis**								
Shulman 2012	94.44	63.89	91.15	95.83	39.58	97.92	0.89	Y
**Rhinosinusitis**								
Blomgren 2005	86.11	55.56	32.81	94.44	10.42	16.67	0.91	YM
Esposito 2008	90.28	33.33	72.92	98.61	34.38	83.33	0.89	YM
Chow 2012	90.28	63.89	72.40	98.61	83.33	87.50	0.91	Y
Wald 2013	94.44	79.17	81.77	94.44	56.25	89.58	0.76	Y
**Influenza**								
Bellamy 2006	86.11	56.94	84.38	95.83	60.42	81.25	0.78	Y(3Y,1YM)
Bautista 2009	93.06	61.11	72.40	95.83	55.21	77.08	0.83	Y(3Y,1YM)
Harper 2009	95.83	61.11	78.65	94.44	58.33	89.58	0.92	Y
Morciano 2009	76.39	45.83	70.31	95.83	54.17	58.33	0.84	Y(3Y,1YM)
**Otitis media**								
Takahashi 2012	86.11	77.78	61.98	90.28	25.00	8.33	0.83	YM
Lieberthal 2013	93.06	61.11	90.10	94.44	70.83	72.92	0.84	Y(3Y,1YM)
Bain 2003	84.72	62.50	76.04	95.83	66.67	62.50	0.76	Y(3Y,1YM)
**URTI** 2								
Snellman 2013	97.22	83.33	83.33	94.44	72.92	95.83	0.77	Y(3Y,1YM)
**Medium (range)**	90.28 (76.39–97.22)	61.11 (33.33–83.33)	76.04 (32.81–91.15)	95.83 (90.28–98.61)	56.25 (10.42–83.33)	81.25 (8.33–97.92)	/	/

Notes:

1 Y: Yes; YM: Yes, with modifications; N: No.

2 URTIs refer to guidelines which include multiple diseases in URTIs.

### Recommendations

#### Recommendations towards drug therapy of Group A streptococcal pharyngitis for children (Table 4)

Recommendations on antibiotics from IDSA and ISCI are consensus. The first choice was penicillin or amoxicillin. For penicillin-allergic patients, cephalosporins, clindamycin, or macrolides were recommended. Only IDSA, however, gave a recommendation on the duration of antibiotics (10 days). In terms of adjunctive therapy, IDSA suggested nonsteroidal anti-inflammatory drugs (NSAIDs) as adjunct to an appropriate antibiotic for treatment of moderate to severe symptoms or control fever. Aspirin should be avoided in children due to the risk of Reye’s syndrome [28].

**Table 4:** Main Therapeutic Options on Group A Streptococcal Pharyngitis for Children According to Guidelines.

Therapy recommended	Shulman 2012,IDSA	Snellman 2013,ISCI
**Target of population**	Children >3 years	Children
**Antibiotics**		
** Onset of antibiotics**	Diagnosis of pharyngitis	Culture positive cases of group A streptococcal pharyngitis
** Type of antibiotics**		
** First-line**	Penicillin, amoxicillin (strong, high)	Penicillin, amoxicillin
** Second-line (penicillin allergy)**	A first-generation cephalosporin^1^, clindamycin, clarithromycin, azithromycin (strong, moderate)	Cephalosprins^1^, macrolides, clindamycin, amoxicillin- clavulanate (2 low quality studies; 1 high quality study)
** Duration**	10 days^2^	–
**Adjunctive drugs**		
** NSAIDs** 3	For treatment of moderate to severe symptoms or control fever (high, strong)	–
** Corticosteroids**	NR^4^ (moderate, weak)	–

Notes:

1 The first-generation cephalosporins can be used for patients who are not anaphylactically sensitive.

2 Azithromycin should be given for 5 days.

3 NSAIDs: nonsteroidal anti-inflammatory drug.

4 NR: not recommended.

#### Recommendations towards drug therapy of acute bacterial rhinosinusitis (ABRS)/sinusitis for children (Table 5)

All of the 5 guidelines supported the use of antibiotics in pediatric ABRS. However, AAP emphasized the onset of antibiotics should be in cases of severe onset or worsening course, while the other four recommended antibiotics for all clinical diagnosed ABRS. Guidelines from FSP and SIP recommended amoxicillin as first-line choice due to the low risk of treatment failure [10]. IDSA, however, suggested amoxicillin-clavulanate rather than amoxicillin as empiric antimicrobial therapy, considering the increasing prevalence of H. influenza among URTI of children and the high prevalence of β-lactamase-producing respiratory pathogens in ABRS [29], [30]. For children with risk factors, amoxicillin-clavulanate was recommended. For non-type 1 hypertension, both ISCI and IDSA recommended doxycycline as an alternative for older children. Nevertheless, a variance appeared in terms of antibiotics for type 1 hypertension patients. ISCI and IDSA suggested levofloxacin, while AAP recommended cephalosporins based on recent studies which indicated the risk of a serious allergic reaction to cephalosporinsin patients with penicillin or amoxicillin allergy appeared to be nil [31]–[33]. The duration of antibiotic therapy is still controversial (3–28) [34]. In addition, all guidelines consistently deprecated decongestants, antihistamine and systemic corticosteroids (not local corticosteroids) in pediatric ABRS.

**Table 5:** Main Therapeutic Options on Acute Bacterial Rhinosinusitis(ABRS)/Sinusitis for Children According to Guidelines.

Therapy recommended	Blomgren 2005, FSO	Esposito 2008, SIP	Chow 2012, IDSA	Wald 2013, AAP	Snellman 2013, ISCI
**Target of population**	Children >1 year	Children >1 year	Children	Children aged 1–18 years	Children
**Antibiotics**					
** Onset of antibiotics**	Clinical diagnosis of ABRS (B)	Clinical diagnosis of ABRS (I,A)	Clinical diagnosis of ABRS (strong, moderate)	Severe onset or worsening course ABRS (B, strong recommendation)	Clinical diagnosis of ABRS (high quality)
** Types of antibiotics**					
** First-line**	Amoxicillin	**Mild:** Amoxicillin (IV,B)	Amoxicillin-clavulanate (Strong, moderate)	Amoxicillin or amoxicillin- clavulanate (B, Recommendation).	Amoxicillin-clavulanate (high dose may consider in children <2 years) (Guideline).
** Second-line**					
**With risk factors** 1	**–**	**Mild:** Amoxicillin-clavulanic or cefaclor (IV, B) **Severe:** ceftriaxone, amoxicillin-clavulanic, ampicillin-sulbactam (IV, B)	Amoxicillin-clavulanate (weak, moderate) or third-generation oral cephalosporin+clindamycin (weak, moderate).	Amoxicillin-clavulanate	**–**
** Hypersensitivity**					
** Non-type I**	**–**	–	Third-generation oral cephalosporin+clindamycin (weak, moderate), doxycycline (>8 years)	Cefdinr, cefuroxime, cefpodoxime	Doxycycline (for older children), Levofoxacin
** Type I**	**–**	–	Levofloxacin (weak, low).	Cefdinir, cefuroxime, cefpodoxime, or cefixime +clindamycin	
** Durations**	7days	**Mild**: 10–14 days (IV,B) **Severe**: 14–21 days (IV,B)	10–14 days (weak, low-moderate).	10–28 days	3–14 days (Low quality evidence)
**Adjunctive drugs**					
**Corticosteroids**	Recommended to allergic patients	NR (II,A)	Recommended to allergic patients (weak, moderate)	–	Recommended for recurrent or allergic patients (high quality evidence).
**Decongestants**	NR^2^	NR (II,A)	NR(strong, low-moderate)	–	NR
**Antihistamines**	NR	NR (II,A)	NR(Strong, low-moderate)	–	NR

Notes:

1 Risk factors include: previous receive of antibiotic therapy; attendance at school, local or systematic diseases that favor infections due to antibiotic-resistant pathogens; from geographic regions with high endemic rates of invasive penicillin-nonsusceptible (PNS) S. pneumonia; severe infection; age<2; recent hospitalization; immunocompromised.

2 NR: not recommended.

#### Recommendations towards drug therapy of influenza (Table 6)

H1N1: Both IDSA and WHO recommended antivirals for confirmed or highly suspected H1N1 infection. However, IDSA recommended zanamivir rather than oseltamivir, while WHO recommended oseltamivir for children (>1 year) who have severe or progressive clinical illness.

**Table 6:** Main Therapeutic Options on Influenza for Children According to Guidelines.

Therapy recommended	H1N1		H3N2	H5N1		Influenza-like syndrome
	Harper 2009, IDSA	Bautista 2009, WHO	Harper 2009, IDSA	Bellamy 2006, WHO	Harper 2009, IDSA	Morciano 2009, SNLG
**Target of population**	Children>1 year	Children (≤12) and Adolescents(13–18 years)	Children >1 year	Children	Children >1 year	Children
**Antivirals**						
**Onset of antivirals**	Laboratory-confirmed or highly suspected infection (A, II)	Confirmed or strongly suspected infection (Low, Strong)	Laboratory-confirmed or highly suspected infection (A, II)	Confirmed or strongly suspected infection	Laboratory-confirmed or highly suspected infection (A, II)	Post-exposure prophylaxis in non-vaccinated institutionalized patients
**Choose of antivirals**	Zanamivir, adamantine (rimantadine) (A, II)	**Pandemic H1N1 with severe or progressive clinical illness:** oseltamivir (Low, strong) **Uncomplicated pandemic H1N1:** oseltamivir, zanamivir(Low,strong)	Oseltamivir, zanamivir (A-II)	Oseltamivir (strong, very low), zanamivir (≥7 years) (weak, very low).	Oseltamivir, zanamivir (A-II)	Oseltamivir (C/I)
**Antibiotics**						
				**Severe community-acquired pneumonia:** follow guidelines (strong) **Mechanical ventilation:** recommend treatment or prevention of ventilator associated or hospital acquired pneumonia (strong)		**Non-complicated:** NR^1^(E/I) **Influenza-like syndrome-related sore throat:** NR, unless symptoms are complicated by bacterial infections(D/I)
**NSAIs**						
		Aspirin: NR (strong, regulatory warning)				Paracetamol, ibuprofen (B/I)

Notes:

1 NR: not recommended.

H3N2: Only IDSA released a guideline on the treatment of H3N2 and recommended oseltamivir or zanamivir for laboratory-confirmed or highly suspectedH3N2. IDSA also warned that adamantanes should not be used.

H5N1: The recommendations on use of antiviral drugs for H5N1 were based predominantly on studies of infection with human influenza rather than clinical trials on treatment of H5N1 patients. Both WHO and IDSA placed a high value on the prevention of death and relatively low values on adverse reactions, development of resistance, and costs of treatment. Oseltamivir and zanamivir were recommended as first-line therapy and amantadine was recommended when neuraminidase inhibitors were not available.

Influenza-like syndrome: The SNLG did not recommend the routine use of amantadine, rimantadine, oseltamivir or zanamivir for influenza-like syndrome because of their side effects, the emerging resistance phenomena, and the irrelevance of the outcomes considered in the selected studies. Instead, SNLG recommended the use of oseltamivir in the post-exposure prophylaxis in non-vaccinated institutionalized patients.

#### Recommendations towards drug therapy of acute otitis media (Table 7)

SIGN, JOS and AAP all recommended an observation of 2–3 days before antibiotic therapy for mild AOM. AAP recommended amoxicillin as the first choice and an antibiotic with additional β-lactamase for children with risk factors. SIGN and JOS recommend amoxicillin, amoxicillin-clavulanic, cephalosporins and macrolides with a statement that cephalosporins and macrolides can be used but less safe than amoxicillin [35]. Duration of antimicrobial therapy is still controversial [36]. SIGN recommended a 5 day course according to British National Formulary, while AAP recommended a 5 to 10 day course based on the age of children [35], [37]. Further, only SIGN evaluated the efficacy and safety of adjunctive therapies [38].

**Table 7:** Main Therapeutic Options on Acute Otitis Media (AOM) for Children According to Guidelines.

Therapy recommended	Bain 2003, SIGN	Takahashi 2012, JOS	Lieberthal 2013, AAP
**Target of population**	Children	Children <15 years	Children aged 6 months-12years
**Antibiotics**			
** Onset of antibiotics**	**Mild**: observation for 3 days without use of antimicrobial agents (1+,B)	**Mild**: observation for 3 days without use of antimicrobial agents (A)	**Mild AOM in children (>2 years)** antibiotic therapy or observation for 2–3 days (B, Recommendation) **Mild bilateral AOM in children (6–23 months):** antibiotic therapy (B, Recommendation). **Mild unilateral AOM in children (6–23 months):** antibiotic therapy or observation for 2–3 days (B, Recommendation). **Severe:** antibiotic therapy (B, Strong Recommendation).
** Types of antibiotics**	Amoxicillin, amoxicillin-clavulanic, cefaclor, cotrimoxazole, trimethoprim, erythromycin (1+,B)	Amoxicillin, amoxicillin-clavulanate, ampicillin, cefditoren, ceftriaxone (A)	**First-line** 1 **:** amoxicillin (B,Recommendation). **Second-line** 2: an antibiotic with additional β-lactamase (C, Recommendation).
** Duration**	5 days (1+, B).	–	<2 years: 10 days; 2–5 years: 7 days; >6 years: 5–7 days
**Adjunctive**			
**decongestants**	NR^3^ (1++, A)	–	–
**antihistamines**	NR (1++, A)	–	–
**paracetamol**	Recommended for analgesia^4^ (1+, D)	–	–

Notes:

1 The child does not received amoxicillin in the past 30days or does not have concurrent purulent conjunctivitis or the child is not allergic to penicillin.

2 The child has received amoxicillin in the last 30 days or has concurrent purulent conjunctivitis, or has a history of recurrent AOM unresponsive to amoxicillin.

3 NR: Not Recommended.

4 Parents should give paracetamol for analgesia but should be advised of the potential danger of overuse.

## Discussion

### Variation of Evidence and Recommendation Grading System

CTFPHE was first published in 1979 by Canadian Ministry of Health. The quality of evidence is based on the study design and the strength of recommendation depends on sufficiency of evidence. CTFPHE was the first grading system developed and is the foundation of many other grading systems. However, the CTFPHE still has some drawbacks [27]. For instance, a lack of strong relevance between quality of evidence and strength of recommendation and lack of consideration of results exist consistently among studies [39]. Consequently, many other organizations developed their own grading systems [40], [41]. SIGN grading system developed by the Scottish Intercollegiate Guidelines Network is one of the most-widely used systems [26]. However, it still failed to consider the consistency and indirectness of study results. In 2000, GRADE working group was founded based on organizations from 19 countries including WHO. The aim of this group is to develop a consolidated grading system for quality of evidence and strength of recommendation. In 2004, the first edition of this grading system was published and was recognized by more than 30 organizations including WHO and Cochrane Collaboration. Although the 13 guidelines included in our study were published after 2005, only four of them use the GRADE system. We suggest further guidelines use a comparable uniform grading system to evaluate the quality of evidence and strength of recommendations.

### Quality of Guidelines

The potential benefits of guidelines are only as good as the quality of the guidelines themselves. Appropriate methodologies and rigorous strategies in the guideline development process are important for the successful implementation of the resulting recommendations [7]. For these 13 guidelines, two domains are the main problems which decrease the quality and reliability of guidelines. The first is a failure to seek patients’ views and preferences or fail to report this information. Many methods can be used to consider patients’ expectations such as: formal consultations with patients, participation of patients on guideline development group or external review group, or a literature review of patients’ values. However, these processes were seldom performed or described in guideline development or the final reports of guidelines. This problem is also found in other disease guidelines [42], [43], [44]. The second problem is a lack of consideration of applicability of guidelines. How to facilitate the application of guidelines is as important as how to develop a high quality guideline. The facilitators and barriers that may impact the application of guidelines should be considered when developing the guideline. Also, there is a need to consider how to disseminate and implement the guideline effectively using additional materials such as a quick reference guide, educational tools and patient leaflets. These factors are important but often ignored by guideline developers. Studies on the effectiveness of clinical guideline implementation strategies showed that successful guideline implementation strategies should be multifaceted, and actively engage clinicians throughout the process [45], [46]. Thus, future guidelines should pay more attention to the implementation process of guidelines.

### Factors Contributing to Inconsistencies of Guidelines

Although an important level of consensus appears throughout the various guidelines, there are still some conflicts in recommendations for drug choice and durations of therapy. There are three main reasons contributing to the variances. First, the geographic difference leads to the variance of pathogens and its drug resistance. Second, the recommendations of guidelines were based on different evidence. Recent studies may overturn the results of previous studies. Thus, the timely updated guidelines are more reliable. Third, the expectation and preference of guideline developers and patients may influence the final recommendation. Therefore, a local guideline is more useful for health professionals if there is a conflict among guidelines.

### Suggestions for Future Research

The durations of antimicrobial therapy for rhinosinustis and acute otitis media are still controversial. More studies are needed to compare the different durations of antibiotics in children. In addition, the antivirials for influenza also lack direct evidence. Many recommendations are based on indirect evidence. Thus, more clinical trials or prospective observational studies are needed.

## Conclusions

Future guidelines should use a consistent grading system for quality of evidence and strength of recommendations and seek the preference of stake holders to improve the applicability of guidelines. Further, there are still some areas in pediatric URTI that need more research.

## Supporting Information

Checklist S1 (DOC)
